# A Case of Ultrasound Diagnosis of Fetal Hiatal Hernia in Late Third Trimester of Pregnancy

**DOI:** 10.1155/2015/194090

**Published:** 2015-04-23

**Authors:** Stefania Di Francesco, Mariano Matteo Lanna, Marcello Napolitano, Luciano Maestri, Stefano Faiola, Mariangela Rustico, Enrico Ferrazzi

**Affiliations:** ^1^Department of Obstetrics & Gynecology, Vittore Buzzi Children's Hospital, Via Castelvetro 32, 20154 Milan, Italy; ^2^Department of Radiology and Neuroradiology, Vittore Buzzi Children's Hospital, Via Castelvetro 32, 20154 Milan, Italy; ^3^Pediatric Surgery Unit, Vittore Buzzi Children's Hospital, Via Castelvetro 32, 20154 Milan, Italy

## Abstract

Congenital hiatal hernia is a condition characterized by herniation of the abdominal organs, most commonly the stomach, through a physiological but overlax esophageal hiatus into the thoracic cavity. Prenatal diagnosis of this anomaly is unusual and only eight cases have been reported in the literature. In this paper we describe a case of congenital hiatal hernia that was suspected at ultrasound at 39 weeks' gestation, on the basis of a cystic mass in the posterior mediastinum, juxtaposed to the vertebral body. Postnatal upper gastrointestinal tract series confirmed the prenatal diagnosis. Postnatal management was planned with no urgency. Hiatal hernia is not commonly considered in the differential diagnosis of fetal cystic chest anomalies. This rare case documents the importance of prenatal diagnosis of this anomaly for prenatal counseling and postnatal management.

## 1. Introduction 

Congenital hiatal hernia (CHH) is characterized by the herniation of the abdominal organs, most commonly the stomach, through a physiological but overlax gastroesophageal junction into the thoracic cavity. The prenatal diagnosis of CHH is unusual and in the literature only eight case reports of prenatal diagnosis of this anomaly have been reported [1–5].

The CHH is not commonly considered in the differential prenatal diagnosis of cystic chest anomalies. In this paper we report a case of hiatal hernia, suspected in the late third trimester of gestation, and present a review of the literature on the subject.

## 2. Case Report

**Figure 1:**
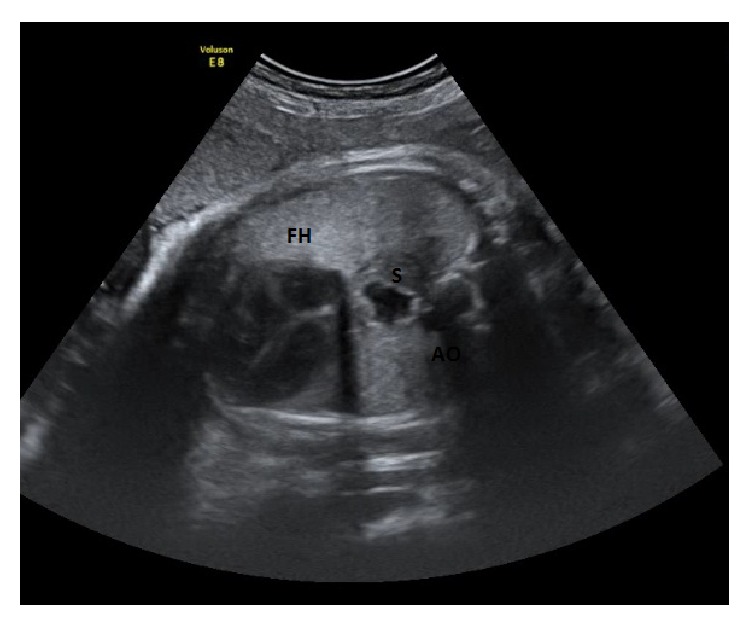
Transverse view of the fetal thorax with an anecoechogenic mass behind the fetal heart (FH). Ao: aorta; S: stomach.

**Figure 2:**
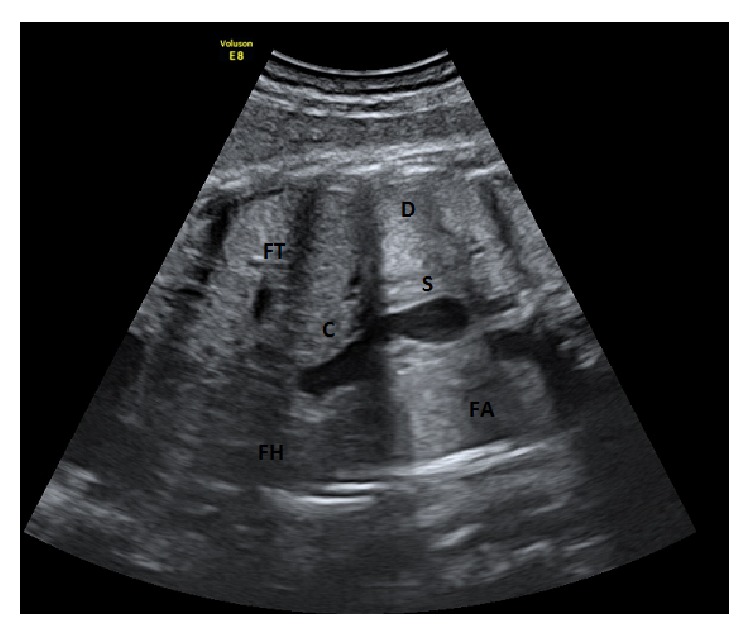
Frontal view: the thoracic cyst (C) is in continuity with a small stomach (S). FH: fetal heart; FA: fetal abdomen; FT: fetal thorax.

A 37-year-old woman (gravida 2, para 1) was referred to our fetal unit at 39 weeks' gestation for evaluation of what appeared to be a small cystic mass in the fetal thorax. The pregnancy had been otherwise uneventful. Family history was unremarkable. Amniocentesis for karyotyping had been performed at 16 weeks for advanced maternal age and revealed a normal karyotype (46, XY). According to Italian guidelines, the patient had undergone ultrasound examinations at 20 and 32 weeks' gestation, and the stomach was normally seen located on the left in the abdomen. Routine ultrasound examination performed at 39 weeks showed a fetus with normal growth. The ultrasound examination was performed because, in our hospital, all patients are evaluated with a clinical and ultrasound examination at 39 weeks of gestation. On the transverse section of the thorax the heart appeared normally located, without mediastinal shift, and the lungs appeared to be of normal echogenicity and volume. A round cystic image, diameter 8 mm, was visible behind the heart in the posterior mediastinum (Figure 1); in sagittal section it appeared to be in continuity with a small stomach located below the diaphragm (Figure 2). During the ultrasound exam period, no modification of the cystic structure was detected. One possible diagnosis was hiatal herniation of the stomach into the thorax and counseling about the diagnostic suspicion of hiatal hernia, the differential diagnosis, and the likely postnatal complications was performed.

**Figure 3:**
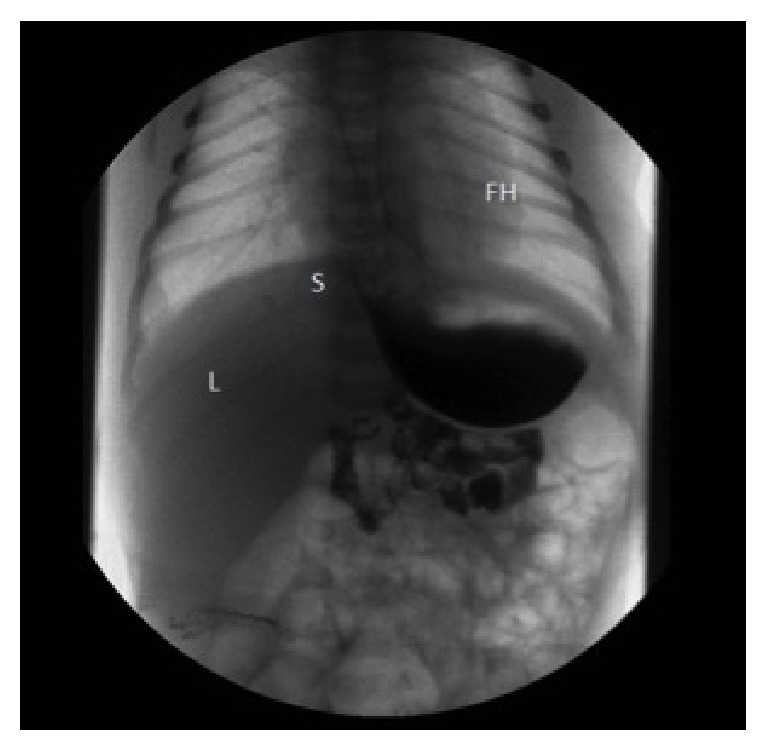
The anteroposterior image of the upper gastrointestinal tract series using barium demonstrating that the stomach (S) was above the diaphragm. FH: fetal heart; L: liver.

At 40 weeks' gestation, the patient was admitted for spontaneous labor. A male newborn, weighing 3460 g, was delivered vaginally with Apgar scores of 9 and 10 at 1st and 5th min, respectively. Postdelivery chest radiography revealed no pulmonary abnormalities and a normal diaphragmatic profile (Figure 3).

The upper gastrointestinal tract series using barium confirmed the prenatal diagnosis of CHH, categorized as type I. During the examination, gastroesophageal reflux was observed, together with a sliding up and down of the stomach into the fetal thorax. The newborn was discharged with prophylactic therapy with ranitidine and domperidon. At six-month follow-up the baby is growing well with sporadic episodes of reflux and he is still on therapy.

## 3. Discussion

Hiatal hernia (HH) is defined as a herniation of the abdominal organs into the chest through the oesophageal diaphragmatic hiatus [3]. This is a well-recognized anomaly in children but most reported cases refer to adult patients. Its etiology is not precisely known, but the hypothesis of its congenital origin is widely accepted. Similarly to other congenital diaphragmatic defects, HH follows a sporadic pattern of incidence in most cases. Familial occurrence of sliding HH has been reported in more than 20 cases, but only one family with two members affected has been described in the literature. The presence of HH in the newborn should alert the clinician to suspect connective tissue disorders such as Ehlers-Danlos syndrome, cutis laxa, and Marfan syndrome [6].

Hiatal hernia often involves parts of the stomach and rarely other abdominal viscera and it is generally classified by the position of the gastroesophageal junction relative to the diaphragm. The gastroesophageal junction is a complex valve composed of a smooth muscle element and a diaphragmatic element. These normally supplement each other to maintain competence in a static condition and during dynamic stress associated with increased intraabdominal pressure [7]. Hiatal hernia is classified as follows: type I, which is a sliding hiatus hernia; type II, also called “rolling” or “pure” paraesophageal hernia that may result from primary diaphragmatic defects; type III, which is a complex mix of types I and II, with migration of the gastroesophageal junction up into the thorax through the hiatus and an additional paraesophageal component; and type IV, essentially a large type III hernia, which may contain the entire stomach, omentum, or other abdominal viscera [6]. Prenatal diagnosis of HH is unusual and to our knowledge only eight cases have been reported in the literature [1–5].

In all reported cases of CHH, the condition was identified in the third trimester of gestation. This late onset may be due to the fact that the fetus may be unable to develop sufficient pressure to dilate the intrathoracic stomach until the third trimester. In the third trimester, the development of adequate pressure during swallowing, probably with the effect of reflux of gastric secretions into the esophagus, could result in visualization of the dilated esophagus/stomach at ultrasound [4].

In the majority of the reports, CHH was an isolated anomaly [1, 3, 4]. However, Yamamoto et al. describe a prenatal diagnosis of type II CHH associated with asplenia syndrome (congenital absence of the spleen, dextrocardia, atrioventricular septal defect, pulmonary stenosis, and intestinal malrotation) [5]. In this fetus, the stomach was observed as intermittently herniating into the thoracic cavity. An intermittent gastric herniation was also described by Ogunyemi in a case of CHH associated with polyhydramnios; in this case the presence of hydramnios was probably due to esophageal reflux correlated with stomach obstruction and the differential diagnosis with esophageal atresia was made by the authors [1]. In three cases, including ours, the stomach was normally seen in the abdomen, while in three other fetuses the stomach was completely herniated into the chest and no intraabdominal stomach was visible; one of these fetuses was affected by CHARGE syndrome [2]. In none of the reported cases was CHH associated with mediastinal shift or pleural/pericardial effusion.

The presence of a cystic mass in the fetal thorax should alert the sonographer to consider in the differential diagnosis congenital chest anomalies such as diaphragmatic hernia, macrocystic adenomatoid malformation of the lung, or rarer conditions such as esophageal duplication or neurenteric cyst [4].

The sonographic criteria for the prenatal diagnosis of CHH were first proposed by Bahado-Singh et al. [3] and then refined by Ruano et al. [4]. The criteria suggested are helpful for making the correct diagnosis and are as follows: (1) the presence of a hypoanechoic mass in the posterior mediastinum, just behind the heart and anterior to the vertebral body, corresponds to a herniated stomach; (2) there is neither mediastinal shift nor pleural or pericardial effusion; the stomach can be identified in the abdominal cavity but in a median position or is not visible in the abdomen because of its complete herniation into the fetal thorax; (3) the dynamic aspect of the herniated stomach means its up–down movements through the enlarged hiatal into the thoracic cavity.

Although congenital hiatal hernia is never a critical condition, it cannot be considered entirely free from significant morbidity. CHH does not carry the same risks as those seen in adult cases. Nonetheless, in the infants and children the herniated stomach into the thorax with continuing acid regurgitation can be complicated by peptic ulceration, severe esophagitis, pyloric stenosis, vomiting, gastric volvulus, aspiration pneumonia, and pulmonary fibrosis [8]. Complications are more prevalent in paraesophageal hernias than in sliding hiatus hernias and elective repair is advocated on diagnosis even in asymptomatic patient in order to prevent life-threatening complication and to avoid the significant morbidity and mortality of an emergency operation [8]. Hiatal hernia is a mechanical defect that will progress so that medical therapy is ineffective and pediatric literature supports routine elective repair of the hernia in infants with congenital HH. The principles of repair consist of reduction of the hernia, partial or complete excision of the sac to prevent recurrence, and crucial approximation followed by an antireflux fundoplication [6, 8].

Congenital hiatal hernia is uncommon and it is rarely prenatally diagnosed but it should be included in the differential diagnosis of any chest anomalies sonographically detected because an early diagnosis and an appropriate postnatal management avoid unnecessary morbidity and even mortality.
